# Supplementary material for: resaCPE: a rapid, low-cost colorimetric assay for the detection of carbapenemase-producing Enterobacterales
Source: J Clin Microbiol. 2026 Feb 27;64(4):e01003-25. doi: 10.1128/jcm.01003-25 (PMC13059814; doi:10.1128/jcm.01003-25)
Supplement: Supplemental materials — Supplemental figures, tables, and methods. [file jcm.01003-25-s0001.docx]

**Supporting Information**

**resaCPE: A Rapid, Low-Cost Colourimetric Assay for the Detection of Carbapenemase-Producing Enterobacterales**

Mitchell A. Jeffs^1^, Gabhan Chalmers^2^, Henry Wong^3,4^, Prameet M. Sheth^1,3,4^, Nicole Ricker^2^ and Christopher T. Lohans^1*^

^1^Department of Biomedical and Molecular Sciences, Queen’s University, Kingston, Ontario, Canada

^2^Department of Pathobiology, University of Guelph, Guelph, Ontario, Canada

^3^Department of Pathology and Molecular Medicine, Queen’s University, Kingston, Ontario, Canada

^4^Division of Microbiology, Kingston Health Sciences Center, Kingston, Ontario, Canada

*Corresponding author: Christopher T. Lohans, [christopher.lohans@queensu.ca](mailto:christopher.lohans@queensu.ca)

Contents

[Supplementary Methodology 3](#_Toc212540499)

[Optimization of lysis conditions for the resaCPE assay 3](#_Toc212540500)

[Optimizing the length of time that the imipenem disk is incubated in the test strain lysate and indicator suspension prewarming time 3](#_Toc212540501)

[Optimizing the volume of indicator strain added to the assay 4](#_Toc212540502)

[Imipenem UV-Vis hydrolysis assays 5](#_Toc212540503)

[Ceftazidime-avibactam disk diffusion assays 5](#_Toc212540504)

[Figure S1: Optimization of lysis conditions for the resaCPE assay. 6](#_Toc212540505)

[Figure S2: Optimizing the length of time that the imipenem disk is incubated with test strain lysates, the indicator suspension prewarming time, and the indicator strain volume used. 7](#_Toc212540506)

[Figure S3: Proof-of-concept experiment for the resaCPE assay employing *E. coli* strains transformed with pACYC184 plasmids carrying genes encoding for carbapenemases (NDM-1, VIM-2) and the penicillinase TEM-116. 8](#_Toc212540507)

[Table S1: Summary of the resaCPE, CARBA-NP and mCIM results for the clinical CPE isolates that were not genomically characterized. 9](#_Toc212540508)

[Table S2: Summary of the resaCPE, CARBA-NP and mCIM results for the genomically characterized CPE panel. 11](#_Toc212540509)

[Table S3: Summary of CPE test results grouped according to species. 12](#_Toc212540510)

[Figure S4: UV-Vis spectrophotometric assays (297 nm) measuring imipenem hydrolysis by CPE isolates exhibiting weak and strong hydrolytic activity. 13](#_Toc212540511)

[Table S4: Summary of the resaCPE, CARBA-NP and mCIM results for the non-CPE panel. 14](#_Toc212540512)

[Figure S5: UV-Vis spectrophotometric assays (monitored at 297 nm) measuring imipenem hydrolysis by non-CPE isolates (AR0039, AR0044) that tested false-positive with the resaCPE or mCIM tests. 16](#_Toc212540513)

[Figure S6: UV-Vis spectrophotometric assays (297 nm) measuring imipenem hydrolysis by CPE isolates in the presence and absence of avibactam. 17](#_Toc212540514)

[Table S5: Ceftazidime-avibactam disk diffusion test results for CPE isolates. 17](#_Toc212540515)

[References 18](#_Toc212540516)

# **Supplementary Methodology**

# **Optimization of lysis conditions for the resaCPE assay**

*E. coli* ATCC 25922 was cultured on CAMHB agar plates as described in the main text. The following day, 2 x 1 µL loopfuls of *E. coli* ATCC 25922 cells were suspended in various volumes of B-PER (400, 350, 300, 250 or 200 µL) in 5 mL culture tubes. Lysates were mixed by vortexing and a 10 µg imipenem disk was added to each sample. Samples were incubated at room temperature for 30 min. Following this incubation, imipenem disks were transferred to 1 mL of sterile CAMHB supplemented with 200 µL of a 0.15 mg/mL solution of resazurin. To confirm that the resazurin reaction was proceeding as expected, a viability control was prepared in which 950 µL of CAMHB was mixed with 50 µL of a 0.5 MFU indicator strain suspension. This viability control did not receive an imipenem disk. All samples were incubated at 37 °C for 2 h; after this incubation, the tubes were inspected visually for colour change.

A subsequent experiment was conducted to evaluate the efficacy of B-PER (200 µL) at lysing clinical CPE isolates. These test strains were cultured on CAMHB agar plates as described in the main text. This experiment with CPE isolates was performed as described in the preceding paragraph, except only 200 µL of B-PER was tested.

# **Optimizing the length of time that the imipenem disk is incubated in the test strain lysate and indicator suspension prewarming time**

*E. coli* ATCC 25922 and test strains were cultured on CAMHB agar plates as described in the main text. For the first set of experiments where different incubation times were tested, 2 x 1 µL loopfuls of *E. coli* ATCC 25922 or clinical isolates were suspended in 200 µL of B-PER in 5 mL culture tubes, which were mixed by vortexing. To each tube, a 10 µg imipenem disk was added. Next, a mixture of 950 µL sterile CAMHB and 50 µL of a 0.5 MFU indicator strain suspension was prepared and added to 5 mL culture tubes for the negative control, test strains and viability control. Both the lysates and the indicator tubes were incubated at 37 °C for 30, 60 or 90 min. After this incubation, 20 µL of a 500 µg/mL proteinase K solution (prepared in sterile water) was added to each indicator tube (final concentration ~10 µg/mL). The imipenem disks were transferred from the lysate tubes to the corresponding indicator tubes using a sterile 1 µL inoculation loop. Note that the viability control did not receive a disk. Lastly, 200 µL of a 0.15 mg/mL resazurin solution (prepared in sterile water) was added to each tube. These tubes were then incubated at 37 °C for 2 h, and the colour of the solutions was visually inspected.

# **Optimizing the volume of indicator strain added to the assay**

An experiment testing the impact of changes to the indicator strain volume was performed as described in the section directly above, with the following modifications. Mixtures of either 975 µL of sterile CAMHB and 25 µL of a 0.5 MFU indicator strain suspension or 987.5 µL of CAMHB and 12.5 µL of indicator strain were prepared. Lysate tubes containing the imipenem disks and the indicator tubes were incubated for 90 minutes prior to proceeding with the resazurin reaction.

# **Imipenem UV-Vis hydrolysis assays**

Test isolates were cultured on CAMHB agar plates as described in the main text. Imipenem (2 mg/mL) and avibactam (1 mg/mL, where required) stock solutions were prepared in sterile Dulbecco’s phosphate buffered saline (DPBS) [Gibco, (-) magnesium and calcium chloride]. For each test strain, 2 x 1 µL loopfuls of cells were suspended in 200 µL of B-PER and incubated at room temperature for 10 minutes. Lysates were diluted 10X in DPBS and 100 µL of each diluted lysate was added to wells of a clear 96-well plate (Griener Bio-One UV Star, flat bottom) in triplicate. Cell lysates were mixed with 100 µL of imipenem solution (30 µg/mL), or imipenem + avibactam (60 µg/mL + 30 µg/mL) where required. Imipenem hydrolysis was monitored every minute for 30 min at 297 nm using a multimode plate reader (Synergy LX).

# **Ceftazidime-avibactam disk diffusion assays**

Test isolates were cultured on CAMHB agar plates as described in the main text. A suspension of each test isolate was prepared in sterile CAMHB to approx. 0.5 MFU. A sterile cotton swab (Puritan) was dipped into each cell suspension and used to coat the surface of a CAMHB agar plate lacking antibiotic. A 30/20 µg ceftazidime/avibactam disk was placed on the surface of the agar plate using sterile forceps. The plates were grown at 37 °C for 20 h without shaking, and the diameter of the zones of inhibition were measured following this incubation. Isolates with a zone diameter of ≤ 20 mm were classified as resistant, according to interpretation criteria (1).


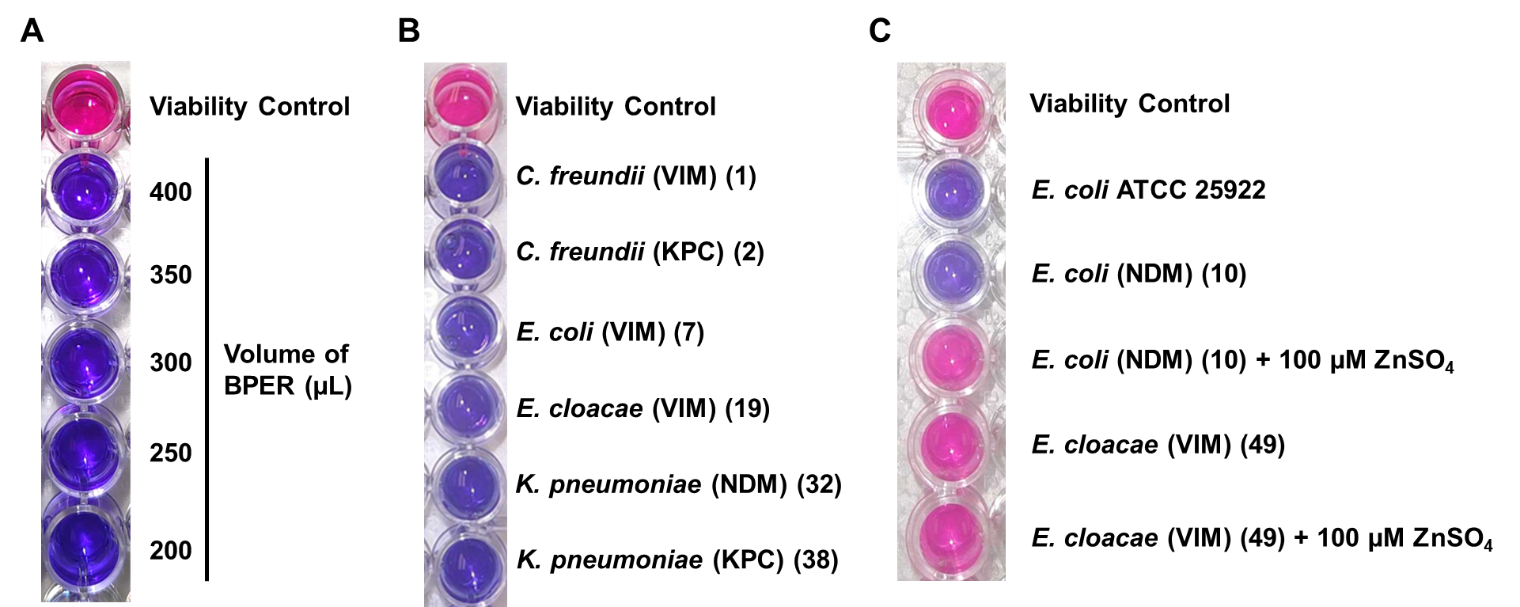


**Figure S1: Optimization of lysis conditions for the resaCPE assay.** (A) Evaluating the efficacy of different volumes of B-PER for lysing *E. coli* ATCC 25922 cells. Imipenem disks were soaked in cell lysates and transferred to a resazurin solution to confirm that any carryover of unlysed cells did not cause a colour change. No colour change was observed for any of the lysis conditions, so 200 µL B-PER was chosen as the lysis volume for future experiments to reduce reagent usage. (B) Resazurin assay results for a range of clinical CPE isolates to test whether B-PER (200 µL) was effective at lysing these strains. No colour change was observed for any of the isolates, which indicated minimal presence of unlysed cells.


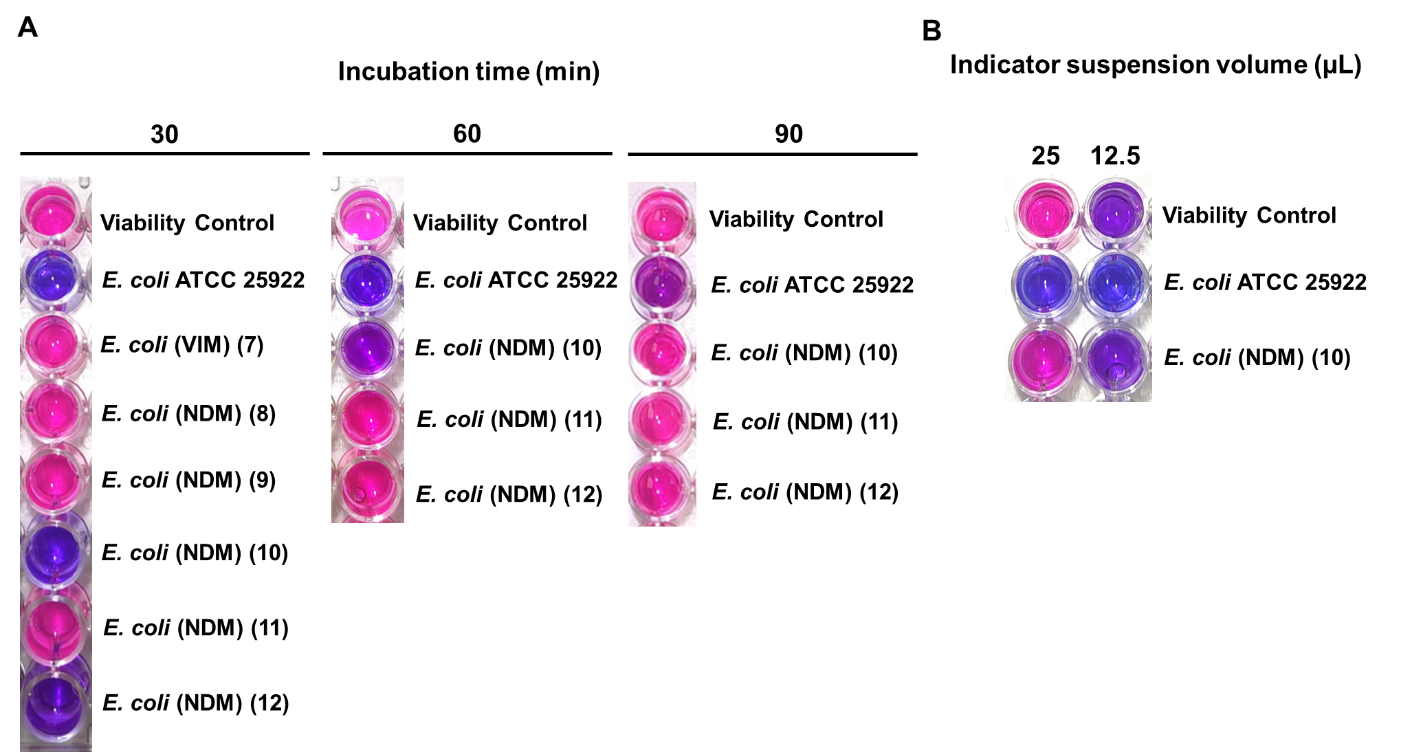


**Figure S2: Optimizing the length of time that the imipenem disk is incubated with test strain lysates, the indicator suspension prewarming time, and the indicator strain volume used.** (A) resaCPE assays conducted with clinical isolates comparing test results when imipenem disks were incubated with test strain lysates for either 30, 60 or 90 minutes at 37 °C prior to the resazurin reaction. Indicator strain suspensions were also incubated at 37 °C for the same length of time. All assays combined 950 µL of cation adjusted Mueller-Hinton broth (CAMHB) with 50 µL of indicator strain suspension prepared to ~0.5 McFarland units (MFU). Isolates 10 and 12 tested false-negative with a 30-minute incubation period but tested positive with a 90-minute incubation. However, with a 90-minute incubation, residual colour change was observed for the sample containing the *E. coli* ATCC 25922 negative control. (B) resaCPE assays resolving the residual colour change in the negative control, using a lower volume of 0.5 MFU indicator strain with a 90-minute incubation period. The desired assay results (*i.e.*, viability control and isolate 10 turned pink, and the negative control remained purple) were obtained for the reactions employing 25 µL of indicator suspension (mixed with 975 µL CAMHB).


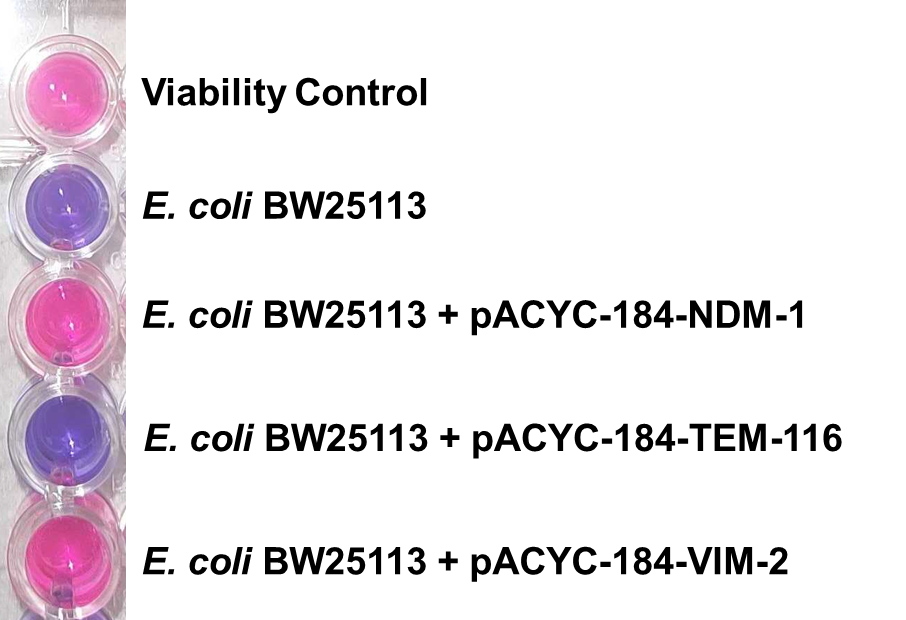


**Figure S3: Proof-of-concept experiment for the resaCPE assay employing *E. coli* strains transformed with pACYC184 plasmids carrying genes encoding for carbapenemases (NDM-1, VIM-2) and the penicillinase TEM-116.** A colour change from purple to pink indicates a positive result for carbapenemase production.

# **Table S1:** Summary of the resaCPE, CARBA-NP and mCIM results for the clinical CPE isolates that were not genomically characterized.

| **Isolate No.** | **Species** | **Carbapenemase** | **resaCPE Result** | **CARBA-NP Result** | **mCIM Result** |
| --- | --- | --- | --- | --- | --- |
| 1 | *C. freundii* | VIM | POS | POS | POS |
| 2 | *C. freundii* | KPC | POS | POS | POS |
| 3 | *C. freundii* | KPC | POS | POS | POS |
| 4 | *C. freundii* | KPC | POS | POS | POS |
| 5 | *C. freundii* | KPC | POS | POS | POS |
| 6 | *C. freundii* | NDM + KPC | POS | POS | POS |
| 7 | *E. coli* | VIM | POS | POS | POS |
| 8 | *E. coli* | NDM | POS | POS | POS |
| 9 | *E. coli* | NDM | POS | POS | POS |
| 10 | *E. coli* | NDM | POS | POS | POS |
| 11 | *E. coli* | NDM | POS | POS | POS |
| 12 | *E. coli* | NDM | POS | POS | POS |
| 13 | *E. coli* | KPC | POS | POS | POS |
| 14 | *E. coli* | KPC | POS | POS | POS |
| 15 | *E. coli* | KPC | POS | POS | POS |
| 16 | *E. coli* | OXA-48-like | POS | NEG | POS |
| 17 | *E. coli* | OXA-48-like | POS | NEG | POS |
| 18 | *E. cloacae* | VIM | POS | POS | POS |
| 19 | *E. cloacae* | VIM | POS | POS | POS |
| 20 | *E. cloacae* | VIM | POS | POS | POS |
| 21 | *E. cloacae* | VIM | POS | POS | POS |
| 22 | *E. cloacae* | VIM | POS | POS | POS |
| 23 | *E. cloacae* | VIM | POS | POS | POS |
| 24 | *E. cloacae* | VIM | POS | POS | POS |
| 25 | *E. cloacae* | VIM | POS | POS | POS |
| 26 | *E. cloacae* | VIM | POS | POS | POS |
| 27 | *E. cloacae* | NDM | POS | POS | POS |
| 28 | *E. cloacae* | NDM | POS | POS | POS |
| 29 | *E. cloacae* | NDM | POS | POS | POS |
| 30 | *K. oxytoca* | KPC | POS | POS | POS |
| 31 | *K. pneumoniae* | VIM | POS | POS | POS |
| 32 | *K. pneumoniae* | NDM | POS | POS | POS |
| 33 | *K. pneumoniae* | NDM | POS | POS | POS |
| 34 | *K. pneumoniae* | KPC | POS | POS | POS |
| 35 | *K. pneumoniae* | KPC | POS | POS | POS |
| 36 | *K. pneumoniae* | KPC | POS | POS | POS |
| 37 | *K. pneumoniae* | KPC | POS | POS | POS |
| 38 | *K. pneumoniae* | KPC | POS | POS | POS |
| 39 | *M. morgannii* | VIM | POS | POS | POS |
| 40 | *E. coli* | NDM | POS | POS | POS |
| 41 | *E. coli* | NDM | POS | POS | POS |
| 42 | *E. coli* | OXA-48-like | POS | POS | POS |
| 43 | *E. coli* | OXA-48-like | POS | POS | POS |
| 44 | *E. coli* | OXA-48-like | POS | POS | POS |
| 45 | *E. coli* | OXA-48-like | POS | POS | POS |
| 46 | *E. coli* | OXA-48-like | POS | POS | POS |
| 47 | *E. coli* | OXA-48-like | POS | POS | POS |
| 48 | *E. cloacae* | IMI | POS | NEG | POS |
| 49 | *E. cloacae* | VIM | POS | POS | POS |
| 50 | *E. cloacae* | VIM | POS | POS | POS |
| 51 | *K. oxytoca* | KPC | POS | POS | POS |
| 52 | *E. coli* | NDM | POS | POS | POS |
| 53 | *E. coli* | KPC | POS | POS | POS |
| 54 | *K. pneumoniae* | KPC | POS | POS | POS |
| 55 | *K. pneumoniae* | KPC | POS | POS | POS |
| 56 | *E. coli* | KPC | POS | POS | POS |
| 57 | *K. oxytoca* | KPC | POS | POS | POS |
| 58 | *E. cloacae* | IMI | POS | POS | POS |
| 59 | *M. morgannii* | KPC | POS | POS | POS |
| 60 | *C. freundii* | KPC | POS | POS | POS |
| 61 | *C. freundii* | KPC | POS | POS | POS |
| 62 | *K. pneumoniae* | KPC | POS | POS | POS |
| 63 | *E. coli* | KPC | POS | POS | POS |
| 64 | *K. pneumoniae* | KPC | POS | POS | POS |
| 65 | *K. pneumoniae* | KPC | POS | POS | POS |

# **Table S2:** Summary of the resaCPE, CARBA-NP and mCIM results for the genomically characterized CPE panel.

| **Isolate No.** | **Species** | **Carbapenemase** | **resaCPE Result** | **CARBA-NP Result** | **mCIM Result** |
| --- | --- | --- | --- | --- | --- |
| AR0034 | *K. pneumoniae* | IMP-4 | POS | POS | POS |
| AR0051 | *K. ozaenae* | OXA-181 | POS | NEG | POS |
| AR0066 | *K. pneumoniae* | OXA-232 | POS | NEG | NEG |
| AR0074 | *E. aerogenes* | OXA-48 | POS | NEG | POS |
| AR0075 | *K. pneumoniae* | OXA-232 | POS | NEG | NEG |
| AR0080 | *K. pneumoniae* | IMP-4 | POS | POS | POS |
| AR0091 | *S. marcescens* | SME-3 | POS | POS | POS |
| AR0099 | *S. marcescens* | SME-3 | POS | POS | POS |
| AR0121 | *S. marcescens* | SME-3 | POS | POS | POS |
| AR0122 | *S. marcescens* | SME-3 | POS | POS | POS |
| AR0123 | *S. marcescens* | SME-3 | NEG | POS | POS |
| AR0138 | *K. pneumoniae* | NDM-7 | POS | POS | POS |
| AR0140 | *K. pneumoniae* | OXA-181 | POS | NEG | POS |
| AR0142 | *K. pneumoniae* | OXA-181 | POS | NEG | POS |
| AR0149 | *E. coli* | NDM-7 | POS | POS | POS |
| AR0161 | *E. aerogenes* | IMP-4 | POS | POS | POS |
| AR0162 | *E. coli* | NDM-7 | POS | NEG | NEG |

# **Table S3:** Summary of CPE test results grouped according to species.

| **Species** | **resaCPE Result** | **CARBA-NP Result** | **mCIM Result** |
| --- | --- | --- | --- |
| *C. freundii* | 8 / 8 | 8 / 8 | 8 / 8 |
| *E. aerogenes* | 2 / 2 | 1 / 2 | 2 / 2 |
| *E. cloacae* | 16 / 16 | 15 / 16 | 16 / 16 |
| *E. coli* | 25 / 25 | 22 / 25 | 24 / 25 |
| *K. oxytoca* | 3 / 3 | 3 / 3 | 3 / 3 |
| *K. ozaenae* | 1 / 1 | 0 / 1 | 1 / 1 |
| *K. pneumoniae* | 20 / 20 | 16 / 20 | 18 / 20 |
| *M. morgannii* | 2 / 2 | 2 / 2 | 2 / 2 |
| *S. marcescens* | 4 / 5 | 5 / 5 | 5 /5 |


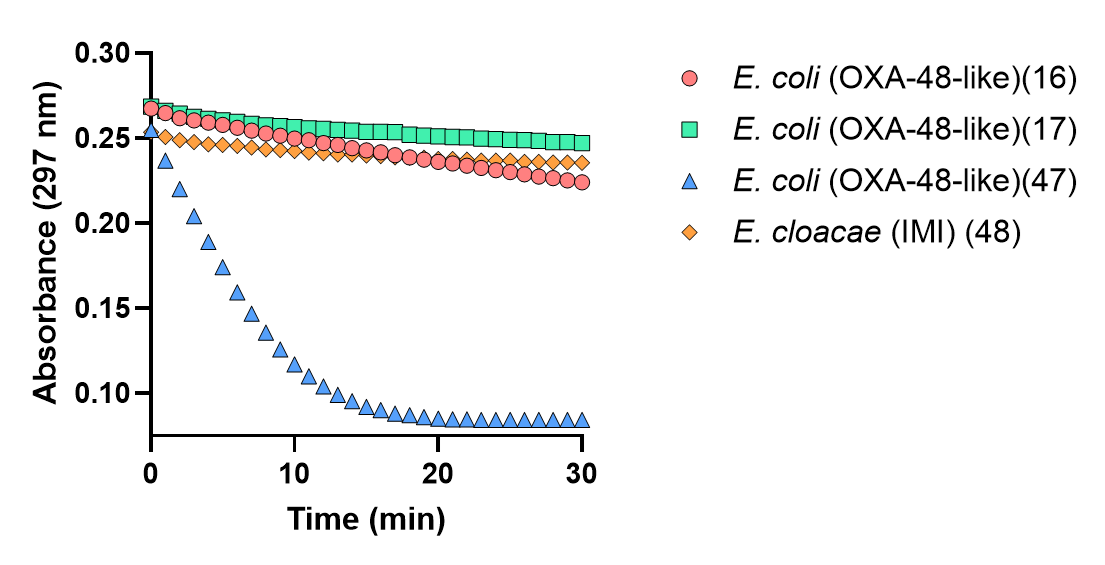


**Figure S4: UV-Vis spectrophotometric assays (297 nm) measuring imipenem hydrolysis by CPE isolates exhibiting weak and strong hydrolytic activity.** Two OXA-48-like and one IMI-producing isolates (numbers 16, 17 and 48) tested false-negative for carbapenemase production via the CARBA-NP test and exhibit weak hydrolytic activity as measured by UV-Vis spectrophotometry. Isolate 47, an OXA-48-like producer, tested positive for carbapenemase via the CARBA-NP, and exhibits faster imipenem hydrolysis. Isolates were tested in triplicate, error bars indicate S.D.

# **Table S4:** Summary of the resaCPE, CARBA-NP and mCIM results for the non-CPE panel.

| **Isolate No** | **Species** | **β-Lactamase** | **resaCPE Result** | **CARBA-NP Result** | **mCIM Result** |
| --- | --- | --- | --- | --- | --- |
| N1 | *P. mirabilis* | CTX-M-15 | NEG | NEG | NEG |
| N2 | *P. mirabilis* | CTX-M-15 | NEG | NEG | NEG |
| N3 | *P. mirabilis* | CTX-M-15 | NEG | NEG | NEG |
| N4 | *P. mirabilis* | CTX-M-15 | NEG | NEG | NEG |
| N5 | *P. mirabilis* | CTX-M-15 | NEG | NEG | NEG |
| N6 | *P. mirabilis* | CTX-M-15 | NEG | NEG | NEG |
| N7 | *P. mirabilis* | CTX-M-15 | NEG | NEG | NEG |
| N8 | *P. mirabilis* | CTX-M-15 | NEG | NEG | NEG |
| N9 | *P. mirabilis* | CTX-M-15 | NEG | NEG | NEG |
| N10 | *P. mirabilis* | CTX-M-15 | NEG | NEG | NEG |
| N11 | *P. mirabilis* | CTX-M-15 | NEG | NEG | NEG |
| N12 | *P. mirabilis* | CTX-M-15 | NEG | NEG | NEG |
| N13 | *E. coli* | TEM-1, CTX-M-55 | NEG | NEG | NEG |
| N14 | *E. coli* | CTX-M-14 | NEG | NEG | NEG |
| N15 | *E. coli* | CTX-M-15 | NEG | NEG | NEG |
| N16 | *E. coli* | TEM-1, CTX-M-1 | NEG | NEG | NEG |
| N17 | *E. coli* | CMY-2, CTX-M-14 | NEG | NEG | NEG |
| N18 | *E. coli* | CTX-M-15 | NEG | NEG | NEG |
| N19 | *E. coli* | CTX-M-15 | NEG | NEG | NEG |
| N20 | *E. coli* | CTX-M-15 | NEG | NEG | NEG |
| N21 | *E. coli* | CTX-M-55 | NEG | NEG | NEG |
| N22 | *E. coli* | CMY-2 | NEG | NEG | NEG |
| N23 | *E. coli* | CMY-2 | NEG | NEG | NEG |
| N24 | *E. coli* | CMY-2, CTX-M-15 | NEG | NEG | NEG |
| N25 | *E. coli* | CTX-M-32 | NEG | NEG | NEG |
| N26 | *E. coli* | CTX-M-14 | NEG | NEG | NEG |
| AR0010 | *K. pneumoniae* | CMY-94, SHV-1 | NEG | NEG | NEG |
| AR0012 | *K. pneumoniae* | SHV-12 | NEG | NEG | NEG |
| AR0014 | *E. coli* | CTX-M-15, OXA-1 | NEG | NEG | NEG |
| AR0015 | *E. coli* | CTX-M-15, OXA-1, TEM-1 | NEG | NEG | NEG |
| AR0039 | *K. pneumoniae* | CTX-M-15, OXA-1, OXA-9, SHV-12, TEM-1 | POS | NEG | POS |
| AR0043 | *K. pneumoniae* | SHV-12 | NEG | NEG | NEG |
| AR044 | *K. pneumoniae* | CTX-M-15, OXA-1, OXA-9, SHV-12 | POS | NEG | NEG |
| AR0087 | *K. pneumoniae* | SHV-12 | NEG | NEG | NEG |


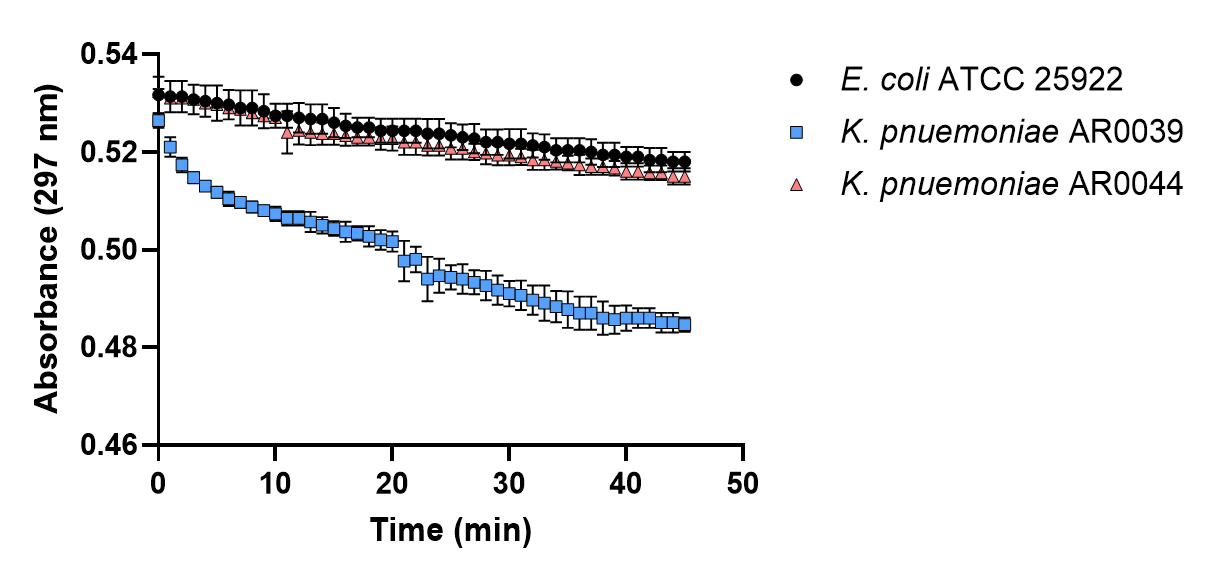


**Figure S5: UV-Vis spectrophotometric assays (monitored at 297 nm) measuring imipenem hydrolysis by non-CPE isolates (AR0039, AR0044) that tested false-positive with the resaCPE or mCIM tests.** Isolates were tested in triplicate, error bars indicate SD.


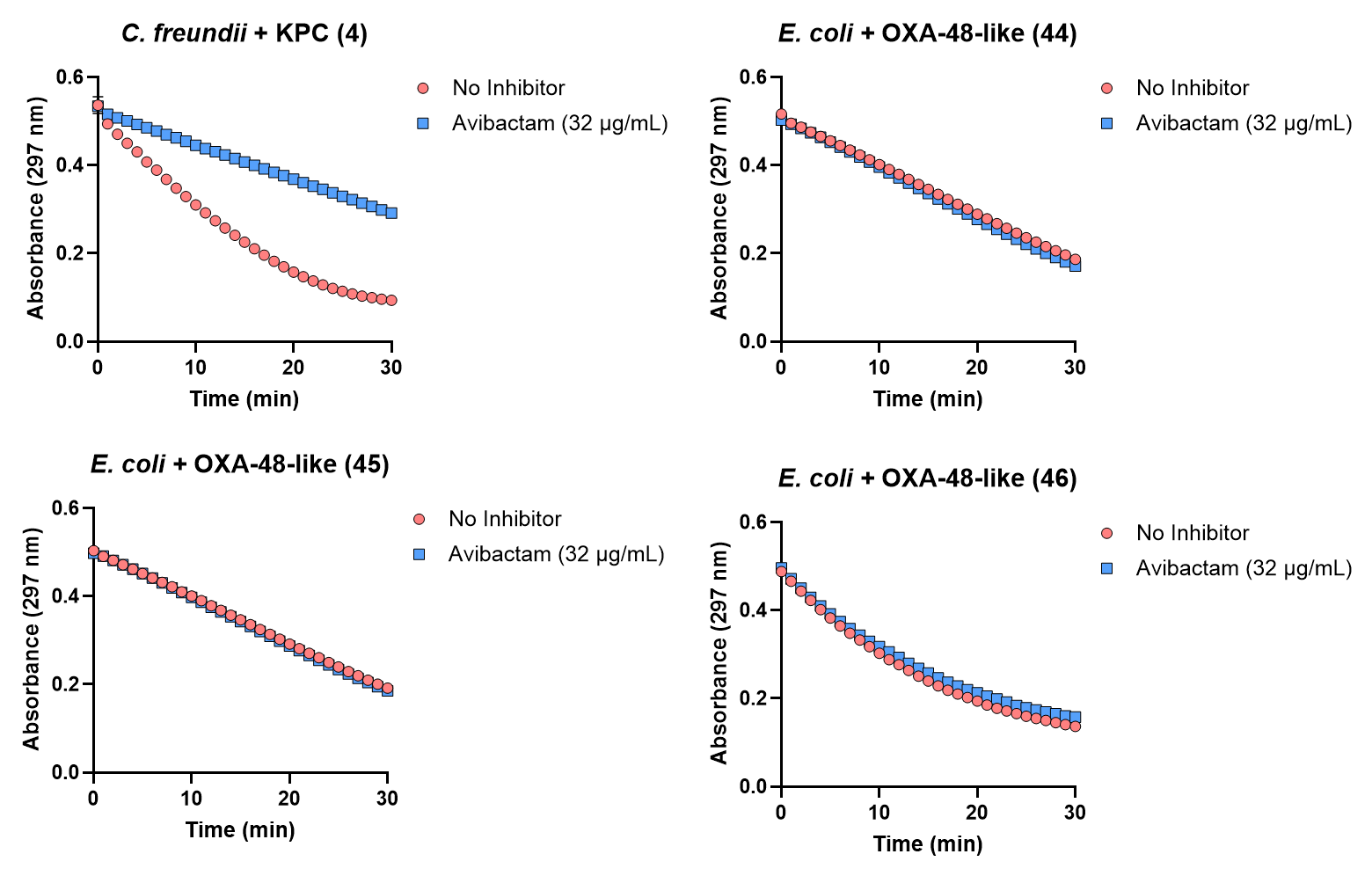


**Figure S6: UV-Vis spectrophotometric assays (297 nm) measuring imipenem hydrolysis by CPE isolates in the presence and absence of avibactam.** In resaCPE assays, these isolates hydrolyzed imipenem in the presence of the carbapenemase inhibitor avibactam, as indicated by a colour change from purple to pink (see main text). UV-Vis experiments employing avibactam corroborate these results, as imipenem hydrolysis was observed even when avibactam is present. Isolates were tested in triplicate, error bars indicate SD.

**Table S5: Ceftazidime-avibactam disk diffusion test results for CPE isolates**. As per interpretation criteria, a zone diameter of ≤ 20mm indicates resistance to the drug combination (1).

| **Isolate No.** | **Ceftazidime-Avibactam Zone Diameter (mm)** | **Result** |
| --- | --- | --- |
| 4 | 25 | Susceptible |
| 44 | 11 | Resistant |
| 45 | 12 | Resistant |
| 46 | 19 | Resistant |

# **References**

1. Han Renru, Shen Siquan, Yin Dandan, Ding Li, Shi Qingyu, Yang Yang, Guo Yan, Wu Shi, Zhi Peiyuan, Zhu Demei, Hu Fupin. 2023. Performance of Ceftazidime-Avibactam 30/20-μg and 10/4-μg Disks for Susceptibility Testing of Enterobacterales and *Pseudomonas aeruginosa*. Microbiology Spectrum 11:e02720-22.
